# Experimental Investigation into the Connection Performance of Reinforcement Sleeves Utilizing MPC Grouting Materials

**DOI:** 10.3390/ma19081661

**Published:** 2026-04-21

**Authors:** Hao Shu, Lu Chen

**Affiliations:** 1 International Institute of Engineering, Changsha University of Science and Technology, Changsha 410114, China; 15806903756@163.com; 2 School of Civil Engineering and Environment, Changsha University of Science and Technology, Changsha 410114, China

**Keywords:** connection performance, reinforcement sleeves, MPC grouting materials, uniaxial tensile test

## Abstract

**Highlights:**

- Uniaxial tensile tests are conducted on reinforcement sleeve connections grouted with modified polyurethane composite (MPC).
- MPC has excellent fluidity and meets the performance and strength criteria for sleeve grouting materials, making it suitable for reinforced sleeve connections.
- Increasing the strength grade and anchorage length of reinforcing bars can effectively improve the ultimate load of reinforced sleeve specimens with MPC grouting material.

**Abstract:**

With the vigorous promotion of the modernization of China’s construction industry, the proportion of prefabricated buildings in new construction projects has increased steadily. Grouted sleeve connection is a mainstream joining method for prefabricated components, and the performance of grouting materials is crucial to connection reliability. In this study, a modified polyurethane composite (MPC) was developed as a novel sleeve grouting material, and seven grouted splice specimens with different steel bar strength grades and anchorage lengths were fabricated for uniaxial tensile tests. The mechanical properties of MPC and the connection performance of specimens were systematically investigated, and the effects of steel bar strength grade and anchorage length on ultimate load, average bond strength, and strain characteristics were quantitatively analyzed. The results show that MPC has excellent fluidity, and its mechanical strengths meet the specified requirements. Increasing steel bar strength grade and anchorage length significantly improves ultimate load: at a 6d anchorage length, the ultimate load of the S600 series (HRB600E) is 44.85% higher than that of the S400 series (HRB400E); extending the S400 series’ anchorage length from 4d to 8d increases ultimate load by 50.61%. Average bond strength decreases with increasing anchorage length (S400-MPC-8d is 24.70% lower than S400-MPC-4d) but increases with higher steel bar strength grade (S600-MPC-6d is 32.37% higher than S400-MPC-6d). The sleeve remains elastic during the test, ensuring safety. Prediction formulas for average bond strength under slip failure were established, with good agreement between predicted and experimental results. For both HRB400E and HTRB600E steel bars, considering safety and installation errors, a critical anchorage length of 8d is recommended for engineering design.

## 1. Introduction

Reinforcement connection plays a crucial role in prefabricated structural concrete construction, with the reinforcement grouting sleeve connection being one of the primary techniques for reinforcement joining in structures such as beam–column and pier–column connections. The grouting sleeve enhances the average bond strength between the reinforcement and the grouting material by restraining the splitting deformation of the internal grout. This average bond strength is critical to the reliability of the connectors and is key to advancing the technology of grouted sleeve reinforcement connections [1].

With the advancement of ecological civilization, the mechanical properties of prefabricated structural joints are subject to higher demands. Factors such as sleeve grouting material, reinforcement strength grade, anchorage length of the connecting reinforcement, and sleeve type all influence the mechanical properties of prefabricated connection members [2,3]. Quantitatively, Ling et al. [4] reported that for Φ25 mm rebars grouted in a sleeve with an inner diameter of 40 mm, the ultimate load increased from approximately 140 kN at an anchorage length of 6d to approximately 180 kN at 10d, with the failure mode transitioning from pull-out to rebar tensile fracture. In the present study, a similar transition was observed at an anchorage length of approximately 7.1d–7.2d for HRB400E and HRB600E rebars, albeit with significantly higher ultimate loads (481.78 kN and 628.28 kN at 8d, respectively) due to the larger rebar diameter (32 mm) and the higher bond efficiency of MPC grout. Several scholars have conducted extensive research on these influencing factors. Li et al. [4,5] proposed a pre-grouting steel bar insertion process as a replacement for the traditional grouting method, which significantly improved the controllability of grouting quality and adaptability to construction errors. By optimizing the end structure of the steel bar and the design of the internal conical section, the connection performance was further enhanced. Yang et al. [6] developed a UHPC-alveolus grouting sleeve for prefabricated tunnels and tested two types of sleeves (wedge-shaped and wedge-shaped four-thread); based on their results, they recommended a minimum anchorage length of 6.5d for steel bar diameters of 25–32 mm and concrete strength grades of C40–C60. Gao et al. [7] used bond test data from the HRB600E high-strength deformed steel bar and grouting material in the existing grouting sleeve as samples and employed ABAQUS for numerical simulation analysis on the mechanical properties of rebar sleeves with ring grooves. The results from both the formula calculation and numerical simulation indicated that the anchorage length for HRB600 should be 8d. Liu et al. [8] investigated bonding performance by varying the rebar diameter (from 16 mm to 32 mm) and bond length (from 4d to 10d); they cast 27 specimens and conducted pull-out tests, showing that high-strength rebars exhibited good bonding with sleeve grouting material. Wang et al. [9] developed a UHPC grouting sleeve with bolts and conducted uniaxial tensile tests on 21 specimens to evaluate the feasibility of the new sleeve design and evaluate the impact of various design parameters. The results demonstrated that the bolt significantly enhanced the anchoring effect of the steel bar in the grouting material, allowing for a reduction in the steel bar anchoring segment length to 0.5d.

Scholars have conducted extensive research on the mechanical behaviors of grouting sleeve connections. Seo et al. [10] investigated the tensile strength of reinforced grouting sleeve joints, developed a connected sleeve (HSS), and explored a method for evaluating its strength. The results indicated that the optimal diameter ratio between the sleeve and the rebar is 1.3. Moreover, the tensile properties of the HSS with a standard size could be accurately predicted using the proposed strength evaluation method. Einea et al. [11] conducted uniaxial tensile tests on four types of fully grouted sleeves; the maximum load-bearing capacity increased with grouting material strength (from 60 MPa to 90 MPa) and rebar anchorage length, and an anchorage length of 7d was found to meet the strength requirements. Zhao et al. [12] systematically tested semi-grouted sleeve connections at ambient temperature (20 °C) and low temperature (−10 °C), using 15 specimens with grouting defects of 0%, 10%, and 20% void ratios; they verified the feasibility of low-temperature grouting technology for winter construction. Wu et al. [13] investigated the impact of the material’s age and the type of rebar on the mechanical behaviors of the specimen. The results showed that the bearing capacity and deformation of the connection increased rapidly during the first week and then stabilized. Yu et al. [14,15] investigated the stress mechanism of rebar sleeve grouting joints through uniaxial tensile tests, examining their damage patterns, bearing capacity, and ductile circumferential strain. Cao et al. [16] investigated specimens with grouting sleeve connections, optimized the mix ratio of the RPC material, and conducted uniaxial tensile tests on these specimens, deriving a model for the average bond strength of the rebar within the sleeve. Furthermore, recent systematic reviews have quantitatively summarized the influencing factors of grouted sleeve connections. Long et al. [17] analyzed over 120 research articles and reported that anchorage lengths ranging from 4d to 12d, rebar diameters from 12 mm to 40 mm, grout compressive strengths from 60 MPa to 120 MPa, and temperatures from −20 °C to 40 °C all significantly affect connection performance. Liu et al. [18] developed a Failure Pattern Identification Method (FPIM) and found that for rebar diameters of 25 mm and 32 mm, the critical embedded length to avoid bond failure is 7.5d at room temperature; elevated temperature (60 °C) reduces the bond strength by approximately 22%. In parallel, efforts have been made to modify polymer-based grouting materials. Zou et al. [19] incorporated discarded rubber particles at dosages of 0%, 3%, 6%, 9%, 12%, and 15% into polyurethane-reinforced cementitious composites with water-cement ratios of 0.65, 0.70, and 0.75; the optimal toughness was achieved at 0.70 water-cement ratio with 6% rubber content, while maintaining compressive strength above 50 MPa and flexural strength above 12 MPa.

Compared with conventional grouting materials such as UHPC and ordinary Portland cement grout that depend on hydration and particle packing, magnesium MPC achieves rapid chemical crosslinking at room temperature to form a dense and tough three-dimensional network, exhibiting obvious advantages of fast curing, high bond strength with reinforcement, and excellent toughness. These characteristics make MPC particularly suitable for rapid repair, emergency reinforcement, and prefabricated component connections requiring fast formwork removal, thus showing great potential as a high-performance grouting material for sleeve connections.

In summary, existing studies have focused extensively on various concrete grout materials and their connection properties. However, the feasibility of using organic composite materials as grout and their effectiveness in anchoring joint connections remains to be explored. In this paper, seven steel bar sleeve joint specimens, filled with MPC as the grout material, were prepared for unidirectional tensile testing to investigate the mechanical properties of their joint connections.

## 2. Mechanical Characterization of MPC

### 2.1. Raw Material and Mix Ratio

The mix ratio of MPC grouting material used in this test is detailed in Table 1, which clearly presents the weight proportion of each component. The MPC grouting material is mainly composed of three components, polyol, polyisocyanate, and cement, with their weight proportions being 25%, 25%, and 50%, respectively (equivalent to a weight ratio of 1:1:2).

### 2.2. Performance Test of MPC

#### 2.2.1. Flexural and Compressive Strength Test of MPC

According to the “Test Procedures for Polymer Modified Cement Mortar (DLT 5126-2001)” [20], three prismatic specimens with sizes of 40 × 40 × 160 mm were made, and the flexural and compressive properties of the MPC grout were tested after curing. The mechanical properties of MPC are shown in Table 2.

#### 2.2.2. Tensile Strength Test of MPC

**Figure 1:**
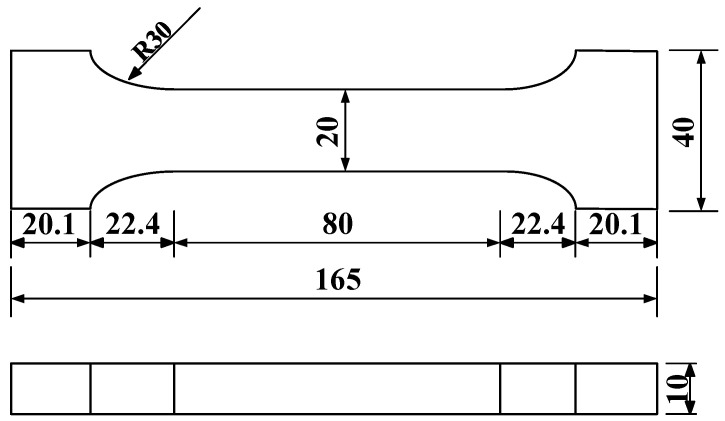
Tensile testing specimen size (mm).

The tensile strength test of MPC was conducted in accordance with the Standard for Test Methods of Physical and Mechanical Properties of Concrete (GB/T 50081-2019) [21]. Dumbbell-shaped specimens were used to perform direct tensile tests. The detailed dimensions of the dumbbell specimens are shown in Figure 1. Three specimens were selected for the tensile strength test, numbered MPC-T1 to MPC-T3 in sequence. A 1000 kN universal material testing machine was used for loading, and an extensometer was adopted to monitor and record the strain development of the specimens during tension. The loading rate was set at 0.5 MPa/s with uniform loading. Test data of the three groups were obtained, the degree of dispersion was analyzed, and the average value was calculated. Overall, the mechanical properties of the modified polyurethane composite (MPC) fully satisfied “Requirements for Sleeve Grouting Materials in Steel Bar Connections (JG/T 408-2019)” [22], and the material also exhibits good market acceptance, making it highly suitable for use as a grouting material in steel bar sleeve connections.

## 3. Connection Performance Test

### 3.1. Specimens Design

**Figure 2:**
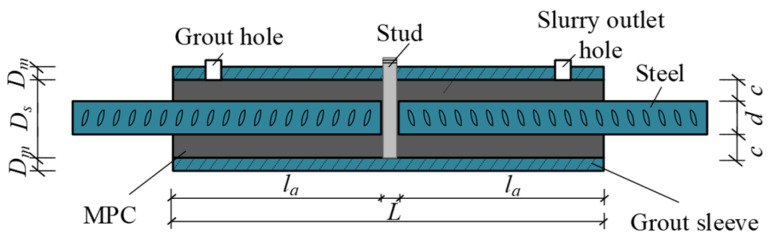
Specimen structure.

In accordance with the relevant test specifications for reinforcement grouting sleeves [23], unidirectional tensile tests were conducted on the specimens, with the rebar anchorage length serving as the experimental variable. The structure and dimensions of the specimens are provided in Figure 2 and Table 3.

### 3.2. Material Properties

**Figure 3:**
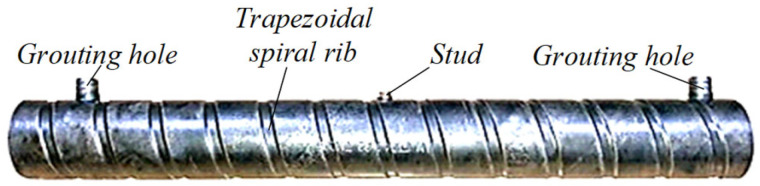
Grouting sleeve.

The grouting sleeve was constructed from high-quality precision seamless steel tube, as shown in Figure 3. The sleeve had a wall thickness of 6 mm, with the outer surface featuring an inverted trapezoidal spiral concave rib, pressed to a depth of approximately 3 mm and with a pitch of 40.8 mm. The rolling process of the outer surface resulted in the formation of spiral convex ribs on the inner surface. This design increased the bonding area between the sleeve and the grouting material, thereby enhancing the average bond strength at the interface.

The HRB400E and HRB600E steel bars with a diameter of 32 mm were chosen for the test. Uniaxial tensile tests of the reinforcement were conducted using a universal testing machine, and the resulting material properties of the reinforcement are presented in Table 4.

### 3.3. Specimen Preparation

The MPC grout was prepared by first pouring polyisocyanate into a container, followed by the gradual addition of cement, fly ash, and other inorganic materials. The mixture was stirred for 5–7 min to ensure uniform mixing. Then, polyol was added and stirred for another 2–4 min until the mixture became homogeneous.

**Figure 4:**
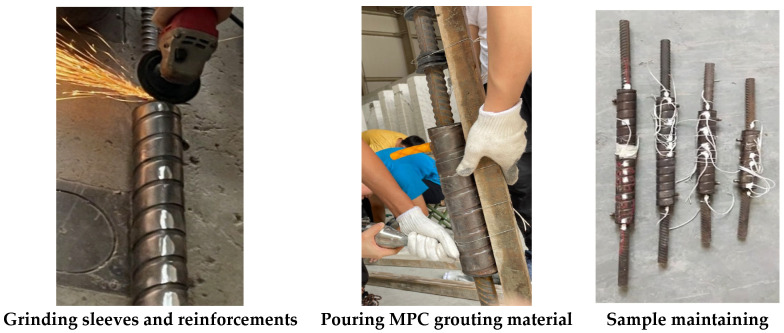
The main fabrication procedure.

The sleeve and steel bar were polished at the positions shown in Figure 4 for strain gauge installation. The connecting steel bar was then inserted into the sleeve from both ends and kept centered. Rubber plugs were installed at both ends of the sleeve before grouting. During grouting, the prepared MPC grout was injected into a grouting gun and manually poured through the grouting hole at the bottom of the sleeve. Grouting was stopped when the grout flowed out from the drainage hole at the top of the sleeve, and the specimen was thus fabricated.

After grouting, the specimen was cured in a standard curing environment for 28 days to ensure satisfactory curing conditions. The main fabrication procedure is illustrated in Figure 4.

### 3.4. Loading and Measurement

**Figure 5:**
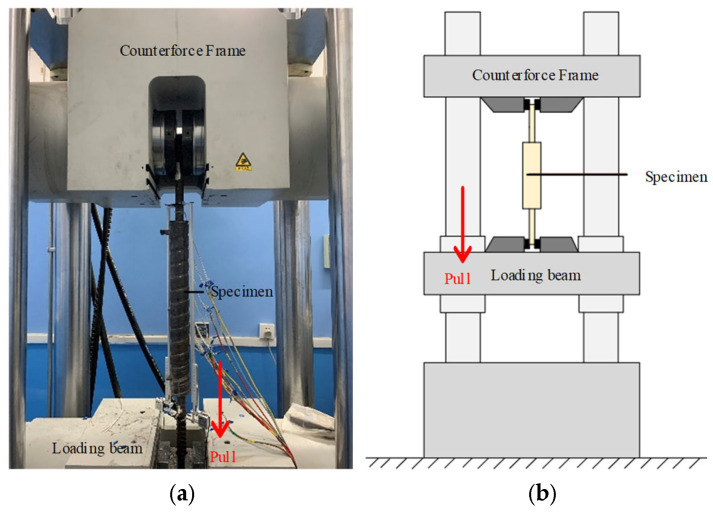
Test loading. (**a**) Actual test diagram; (**b**) test device diagram.

The tensile test of the specimen was carried out on a universal testing machine. During the loading process, the testing machine was initially loaded to the yield point of the specimen and then unloaded to zero to measure the residual deformation. The specimen was subsequently reloaded until failure, with a loading rate of 2 MPa/s. The test setup is illustrated in Figure 5.

**Figure 6:**
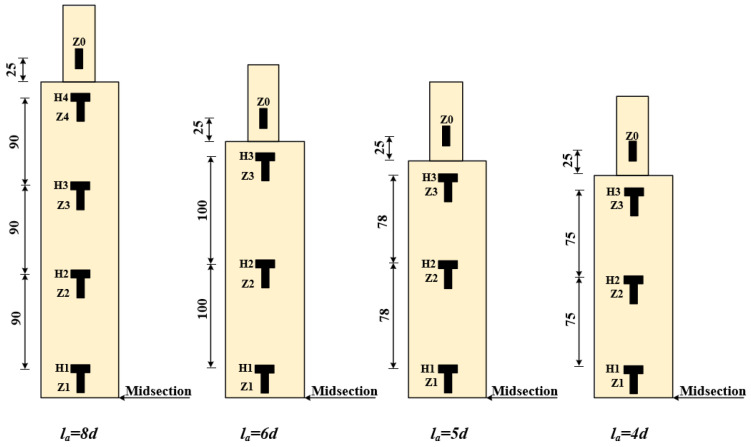
Layout of strain gauges.

During the test, the load and displacement between the fixtures were automatically recorded by the testing machine. Strain gauges were attached to the loading end of the sleeve to observe the strain distribution on the sleeve’s outer surface and the steel bar. The arrangement of the strain gauges on the steel bar and sleeve is shown in Figure 6. “H1–H4” and “Z1–Z4” represent the annular strain gauges and axial strain gauges on the sleeve surface, respectively, while “Z0” represents the axial strain gauge on the steel bar.

The DH3816 static data acquisition instrument was used. The wires connected to the strain gauges were linked to the channels of the instrument, with one wire per channel, using the quarter-bridge connection method. The data acquisition instrument was connected to a computer, which recorded the strain values at each moment throughout the test.

## 4. Test Results and Analysis

### 4.1. Failure Modes

**Figure 7:**
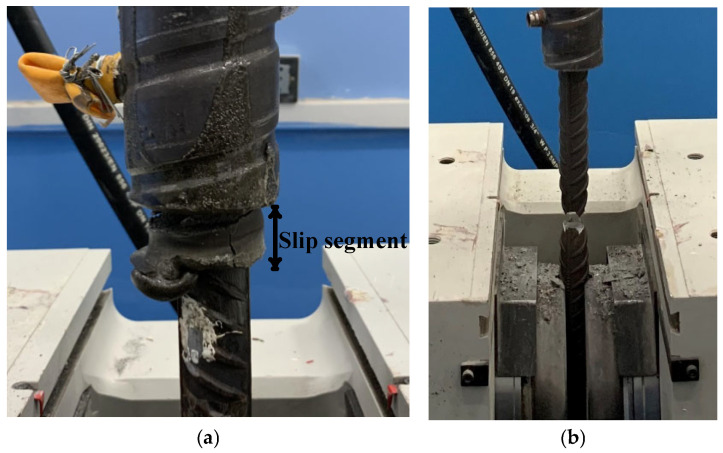
Failure modes. (**a**) Sliding failure of rebar; (**b**) tensile failure of rebar.

There were two failure modes observed during the test: reinforcement tensile fracture and reinforcement slip. Specimens with a length of anchorage segment of the steel bar of 8d experienced tensile failure of the reinforcement, while the others exhibited slip failure, as shown in Figure 7.

For specimens with rebar tensile fracture failure, the grouting material emitted a succession of crisp cracking sounds during loading, indicating the initiation and propagation of its fractures, and eventually fractured into irregular pieces. When the load reached the ultimate bearing capacity of the connecting rebar, the rebar exhibited obvious necking with a substantial reduction in cross-sectional diameter. Eventually, the lower section of the rebar underwent instantaneous tensile fracture at the peak load, which is a typical brittle instantaneous failure mode.

For the specimens exhibiting rebar slip failure, slip initiation occurred as the load increased to exceed the average bond strength between the grouting material and rebar yet did not reach the ultimate tensile strength of the rebar, and the entire failure process was characterized by gradual and progressive development. During tension, the grouting material inside the specimens continuously generated splitting cracks. Owing to the strong confinement imposed by the sleeve wall on the grouting material in the sleeve midsection, no splitting damage occurred in this region. In contrast, the grouting material at the sleeve ends was subjected to reduced restraint, leading to the rapid initiation and propagation of splitting cracks and ultimately causing the failure of the end grouting material. The deformation of the connecting rebar or the failure of the end grouting material induced local slip within the specimens, which resulted in the complete loss of chemical bonding force between the rebar and grouting material at the slip locations. The bond force between the rebar and grouting material consisted primarily of two key components: the frictional resistance at the interface of the grouting material and rebar, and the mechanical interlock between the grouting material and the rebar transverse ribs. When the ultimate bearing capacity of the connecting rebar exceeded the bond force between the rebar and grouting material, the specimens underwent slip failure with a progressive damage pattern.

### 4.2. Load–Displacement Characteristics

**Figure 8:**
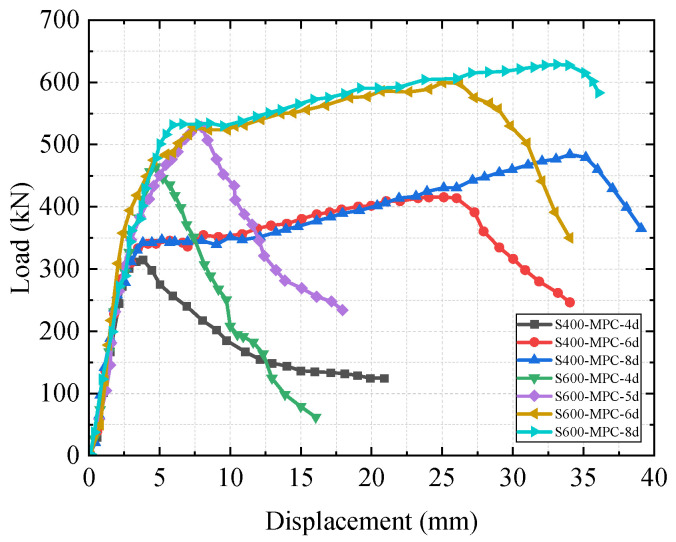
Load–displacement curves.

By applying axial tension to the specimens, the resulting load–displacement curves were obtained, as shown in Figure 8.

The peak load capacities of the specimens were significantly improved by increasing both the strength grade and the length of the anchorage segment of rebars. During the initial loading stage, the steel bars were in the elastic stage. In specimens characterized by an anchorage length of 4d, the interface area between the connecting rebars and the grout material was smaller than that in specimens with anchorage lengths of 6d and 8d, which led to a decrease in bonding force. Slip failure of the steel bars occurred at a smaller displacement in the specimen with an anchorage length of 4d.

Specimens that experienced slip failure exhibited the following characteristics: (1) At the beginning of loading, the rebar was in the elastic stage, demonstrating a proportional relationship between load and displacement, while fine cracks appeared in the internal grouting material. (2) As the load increased, the relative slip between the rebar and the grouting material rose, leading to the continued development of cracks within the grouting material, with more pronounced split cracks occurring at the end of the sleeve. (3) The load further increased and exceeded the adhesion strength between the rebar and the grouting material, causing the rebar to be slowly pulled out, which led to a decrease in load. (4) As displacement increased, the specimen exhibited some circumferential stress due to sleeve constraints, compensating for the loss of circumferential stress experienced before loading. Consequently, the rate of load reduction began to slow down and ultimately stabilized.

Specimens that experienced tensile failure displayed the following characteristics: (1) At the beginning of loading, the ribs of the connecting rebar and the grouting material squeezed against each other, resulting in micro-cracks within the grouting material. There were no cracks at the sleeve end. (2) As the load increased, the rebar began to yield, while the bonding force between the rebar and the material remained greater than the ultimate tensile strength of the rebar. (3) The load continued to rise, and the rebar entered the strengthening stage. Further extrusion occurred between the rebar and the material, causing the existing cracks in the grouting material to develop further, while obvious split cracks emerged at the end of the sleeve. (4) As the load progressively rose, the splitting cracks further developed. Despite this, the adhesion strength between the rebar and material was still greater than the ultimate tensile strength of the rebar, until the rebar entered the neck contraction stage and ultimately broke.

### 4.3. Mechanical Analysis of Specimens

The monotonic axial tensile test was conducted on seven steel bar sleeve connection specimens, with the results summarized in Figure 8 and Table 5. The ultimate load capacities of the specimens S400-MPC-8d and S600-MPC-8d exceeded the ultimate tensile strength of the steel bars, thereby satisfying the criteria for an I-type joint as stipulated in the “Technical Specification for Mechanical Connection of Steel Bars (JGJ 107-2016)” [24]. The ultimate strength of the specimen S600-MPC-6d was greater than the specified characteristic strength but less than 1.10 times of the specified characteristic strength. Additionally, the ratio of fu to the nominal yield strength was greater than 1.25, which conforms to the standards for II-type joint. However, the remaining specimens did not meet the criteria for any of the joint classifications.

Both the steel bar tensile strength grade and the anchorage length have a significant impact on the ultimate load (*P_u_*) of the specimens. A quantitative analysis based on the test data is as follows: The increase in the steel bar tensile strength grade can significantly improve the *P_u_* of the specimens. Under the same anchorage length, the *P_u_* of the S600 series (steel bar strength grade 600) is significantly higher than that of the S400 series (steel bar strength grade 400). Specifically, when the anchorage length is 4d, the *P_u_* of the S600 series is 40.82% higher than that of the S400 series (450.21 kN vs. 319.88 kN); when the anchorage length is 6d, it is 44.85% higher (603.72 kN vs. 416.98 kN); and when the anchorage length is 8d, it is 30.41% higher (628.28 kN vs. 481.78 kN). The effect of anchorage length on *P_u_* is manifested by a significant upward trend of *P_u_* with the increase in anchorage length. In the S400 series, when the anchorage length is increased from 4d to 6d and 8d, the *P_u_* increases from 319.88 kN to 416.98 kN and 481.78 kN, corresponding to an increase of 30.36% and 50.61%, respectively, and further increases by 15.54% when increased from 6d to 8d. In the S600 series, when the anchorage length is sequentially increased from 4d to 5d, 6d, and 8d, the *P_u_* increases from 450.21 kN to 532.84 kN, 603.72 kN, and 628.28 kN, increasing by 18.35%, 34.09%, and 39.47%, respectively. Among them, it increases by 13.30% when increased from 5d to 6d, by 4.02% when increased from 6d to 8d, and by 17.85% when increased from 5d to 8d. Notably, the ratio of *f*_u_ to *f*_yk_ for the S600-MPC-6d specimen is similar to that of the S600-MPC-8d specimen, but unlike the latter, it did not experience tensile failure. This observation indicates that the steel bar anchorage length is a significant factor affecting the failure mode of the specimens, but not the only controlling factor. In contrast, the *f_u_*/*f_y__k_* ratios of specimens S600-MPC-4d and S600-MPC-5d are both below 1.0, and the insufficient anchorage length prevents the HTRB600E high-strength reinforcement from fully exerting its mechanical properties. Overall, the higher the steel bar tensile strength grade and the longer the anchorage length, the greater the ultimate load of the specimens, and the effects of the two show a synergistic effect, which jointly determine the bearing capacity of the specimens.

To further investigate the performance characteristics of magnesium phosphate cement (MPC) as a sleeve grouting material, experimental data of typical ultra-high-performance concrete (UHPC) grouts were collected and are presented in Table 6, and the performance differences between MPC and UHPC in grouted sleeve applications are compared and analyzed.

From the perspective of ultimate bond strength, the MPC grout specimens in this study exhibit a *τ_u_* range of 18.73–35.00 MPa, which is highly competitive with typical UHPC grouts reported in the literature: UHPC specimens tested by Fu et al. [25] show *τ_u_* values of 26.21~35.47 MPa, while those by Zheng et al. [26] range from 16.62~23.34 MPa. Notably, the peak *τ_u_* of 35.00 MPa in the S600-MPC-4d specimen is nearly equivalent to the maximum *τ_u_* of UHPC, and even the minimum *τ_u_* of 18.73 MPa (S400-MPC-8d) exceeds the lower bound of Zheng’s UHPC results, reflecting favorable bond efficiency. In terms of ultimate bearing capacity, when paired with S600-grade high-strength steel bars, the *P_u_* of MPC specimens ranges from 450.21 to 628.28 kN, which is within the same order of magnitude as the UHPC range of 449.11~674.75 kN, with a difference of less than 5%, demonstrating that MPC can effectively transfer steel bar tensile forces and achieve load-bearing capacity comparable to UHPC. Regarding failure modes, both MPC and UHPC follow an identical evolution: at short anchorage lengths (≤6d), interface sliding dominates, indicating grout–steel bond control; as length increases to 8d, both transition to steel bar tensile failure, confirming that MPC bond strength is sufficient to fully develop steel bar tensile strength, aligning with the ideal failure mode for sleeve grouting connections.

The comparison shows that the ultimate bond strength of MPC grout in this study (18.73~35.00 MPa) is comparable to that of typical UHPC grout (16.62~35.47 MPa), and the difference in ultimate bearing capacity from the UHPC group is less than 5%. The evolution of failure modes is consistent with UHPC, and steel bar tensile failure is achieved at long anchorage lengths, indicating that MPC grout can fully utilize the steel bar strength and meet the basic mechanical performance requirements of sleeve grouting connections, showing potential for engineering application as a substitute for UHPC.

### 4.4. Analysis of Interfacial Average Bond Strength Between Rebar and Grout Materials

After passing through the elastic and yield stages, the specimens that experienced slip failure underwent bond slip failure during the strengthening stage, resulting in a sharp decrease in bearing capacity. The actual bond stress surpassed the calculated average bond stress in those specimens that demonstrated tensile failure. For specimens with reinforcement bonding and sliding failure, the average bond strength could be determined using Equation (1):

(1) $$\tau_{u}=\frac{P_{u}}{\pi dl_{a}}=\frac{P_{u}}{\pi nd^{2}}$$

where *d*, *l_a_* and *n* are the diameter, anchorage length, and anchorage multiple of steel bars, respectively.

Based on the test data in Table 5, the quantitative analysis of the effects of steel bar tensile strength grade and anchorage length on the average bond strength (*τ_u_*) of the specimens is as follows: The variation in *τ_u_* presents an opposite law to that of Pu, that is, it decreases significantly with the increase in anchorage length, and under the same anchorage length, the higher the steel bar tensile strength grade, the greater the *τ_u_*. Specifically, for specimens with a reinforcement strength class of HRB400E (S400 series), when the anchorage length is increased from 4d (*τ_u_* = 24.87 MPa) to 6d (*τ_u_* = 21.61 MPa) and 8d (*τ_u_* = 18.73 MPa), *τ_u_* decreases by 13.11% and 24.70%, respectively, and further decreases by 13.33% when increased from 6d to 8d. In the case of specimens with a steel bar strength grade of HRB600E (S600 series), when the anchorage length is sequentially increased from 4d (*τ_u_* = 35.00 MPa) to 5d (*τ_u_* = 33.14 MPa), 6d (*τ_u_* = 31.29 MPa), and 8d (*τ_u_* = 24.42 MPa), *τ_u_* decreases by 5.31%, 14.92%, and 30.23%, respectively (note: the 14.92% decrease from 4d to 6d is the accurate value calculated based on 35.00 MPa to 31.29 MPa); among them, it decreases by 5.61% when increased from 5d to 6d, by 22.02% when increased from 6d to 8d, and by 26.31% when increased from 5d to 8d. This phenomenon of *τ_u_* decreasing with increasing anchorage length occurs because the increased anchorage length enlarges the total area of the reinforced grout bond interface, thereby further enhancing the grout’s grip on the reinforcement and significantly increasing the ultimate load; however, the growth in ultimate load is less than the expansion in bond area, leading to a declining trend in average bond strength. Cross-series comparison shows that when the reinforcement strength grade is increased from HRB400E to HRB600E, the average bond strength is effectively enhanced: for specimens with an anchorage length of 4d, *τ_u_* rises from 24.87 MPa to 35 MPa, representing an increase of 40.73%; for specimens with an anchorage length of 6d, the average bond strength increases by 44.79%, from 21.61 MPa to 31.29 MPa; and for specimens with an anchorage length of 8d, the *τ_u_* of the HRB600E series is 30.38% higher than that of the HRB400E series. This is attributed to the high-strength steel reinforcement bar, which further enhances the mechanical interlock between the rebar and the grout material, significantly increasing the maximum load and markedly elevating the average bond strength. Overall, the increase in steel bar tensile strength grade can effectively improve the interface average bond strength between steel bars and grouting material, while the increase in anchorage length will reduce the average bond strength, which is also related to the uneven growth of ultimate load and bond area.

### 4.5. Strain Analysis of Rebar and Sleeve

**Figure 9:**
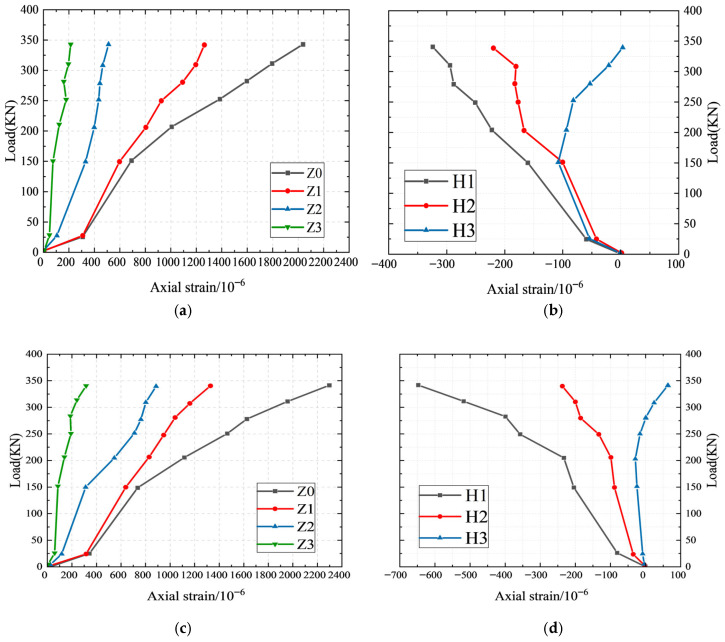
Strain distribution of specimens. (**a**) Axial strain of S400-MPC-6d; (**b**) circumferential strain of S400-MPC-6d; (**c**) axial strain of S600-MPC-6d*;* (**d**) circumferential strain of S600-MPC-6d.

The strain gauge was affixed to both the sleeve and the surface of the rebar to monitor the variations in strain experienced by the specimen throughout the stress testing process. The configuration of the strain gauges is illustrated in Figure 6, while the strain measurements for the S400-MPC-6d and S600-MPC-6d specimens prior to the yielding of the steel bar are presented in Figure 9.

The strain observed in the reinforcement was characterized as tensile strain, which increased linearly in relation to the applied load. Prior to yielding, the strain in the sleeve also exhibited a linear increase, with the strain distribution displaying a characteristic pattern of being maximal at the center and minimal at both ends. Under a uniform load, the absolute values of both axial and circumferential strains on the sleeve surface increased progressively from the ends towards the center of the sleeve. The values of Z3 and H3 at both extremities of the sleeve consistently remained low, while the axial strain Z1 for the S400-MPC-6d specimen ranged from 0 to 1300 με. It was observed that the circumferential strain H3 diminished, with the strain transitioning from compressive to tensile when the load reached 330 MPa. Similarly, the axial strain Z1 for the S600-MPC-6d specimen varied from 0 to 1400 με, with circumferential strain H3 further decreasing. A transition from compressive to tensile strain also occurred in this specimen when the load reached 27 MPa.

The annular strain of the sleeve could be categorized into two principal components. The first component was the tensile strain experienced by the sleeve, which arose from the grouting material subjected to average bond strength exerted by the transverse rib of the rebar. It was observed that as the applied load increased, the bond action intensified, resulting in a correspondingly greater tensile strain. The second component was the compressive strain of the sleeve, which was influenced by the Poisson effect due to tensile forces. This effect engendered compressive strain during the elongation process of the sleeve. The circumferential compressive strain at HS3, located at the terminus of the sleeve, gradually transformed into circumferential tensile strain. This observation indicated that with the progressive increase in the test load, the end grouting material succumbed to average bond strength whilst the sleeve wall experienced compression. The tensile force transmitted from the rebar to the sleeve wall via the grouting material in this region diminished, leading to a situation whereby the tensile strain induced by the sleeve’s extruded expansion surpassed the compressive strain resulting from the Poisson effect. Throughout this scenario, the degree of splitting of the grouting material in the midsection of the sleeve remained minimal, thereby facilitating the complete transfer of longitudinal tensile forces to the sleeve wall. Consequently, the circumferential strain observed at HS2 and HS3 consistently manifested as a compressive strain predominantly governed by the Poisson effect.

Both the longitudinal and circumferential strains of the sleeve exhibited an increase in response to the applied load. The axial tensile strain of the specimen S400-MPC-6d ranged from 0 to 1300 με, while the circumferential compressive strain ranged from 0 to 350 με. In contrast, the axial tensile strain of S600-MPC-6d varied between 0 and 1400 με, with the circumferential compressive strain spanning from 0 to 700 με. Throughout the testing process, the sleeve consistently remained within the elastic phase, satisfying the requisite strength grade and demonstrating a substantial safety margin.

### 4.6. Empirical Formula for Average Bond Strength at Bar–Grout Interface

**Figure 10:**
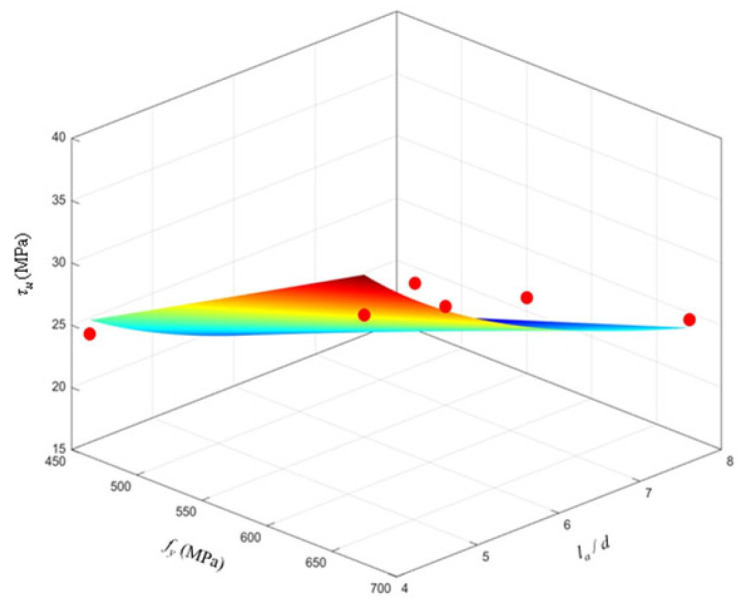
Fitting curves of average bond strength.

As shown in Figure 10, the average bond strength *τ_u_* is related to the compressive strength of MPC, the ratio of the steel bar anchorage length to the steel bar diameter, and the yield strength of the steel bar, as expressed in Equation (2).

(2) $$\tau_{u}=0.275\cdot \left(f_{y}^{1.0304}\right)\cdot \left(f_{c}^{-0.1906}\right)\cdot {\left(\frac{l_{a}}{d}\right)}^{-0.6853}$$

The average bond strengths of specimens that failed by slip were calculated according to Equation (2) and are listed in Table 7. The fitting effect is shown in Figure 10. The mean value, standard deviation, and coefficient of variation in Table 7 are 0.97, 0.07, and 0.08, respectively, indicating that the proposed average bond strength formula fits well and can be used as a design basis for MPC grouted sleeve connections of high-strength steel bars.

Equation (2) is fitted for calculating the average bond strength of HRB400E/HRB600E steel bars (d = 32 mm) anchored in MPC grout (fc = 77.5 MPa) with anchorage lengths of 4d–8d, and it is only applicable to slip failure mode. The formula is not suitable for tensile failure specimens, other grout types, different bar grades/diameters, or anchorage lengths outside 4d–8d. These limitations should be considered in engineering applications.

### 4.7. Critical Anchorage Lengt of Rebar

The critical anchorage length (*L_cu_*) is the anchorage length when the joint rebar is simultaneously subjected to tensile failure and slip failure. Equation (4) can be obtained from the equilibrium condition of specimen failure.

(3) $$\tau_{u}\pi dL_{cu}=Af_{y}$$

where *A* is the cross-sectional area of rebar; ***f*_y_** is the measured tensile strength (MPa) of rebar; and *L_cu_* is the critical anchorage length of rebar (mm). Equation (4) can be deduced from Equation (3). The critical rebar anchorage length of various specimens is presented in Table 7.

(4) $$L_{cu}=\frac{Af_{y}}{\tau_{u}\pi d}=\frac{\pi d^{2}f_{y}}{4\tau_{u}\pi d}=\frac{df_{y}}{4\tau_{u}}$$

When MPC grouting material is used, the critical anchorage lengths for tensile failure of HRB400E and HRB600E steel bars are 7.2d and 7.1d, respectively. Considering safety and the installation error, it is suggested that the critical anchorage length of a connecting steel bar should be 8d.

## 5. Conclusions

This study adopts magnesium phosphate cement (MPC) as a new grouting material for reinforcement sleeve connections and carries out uniaxial tensile tests on seven groups of specimens with HRB400E/HRB600E high-strength rebars. The mechanical behavior, failure mechanism, and bond performance of MPC-grouted sleeve connections are systematically revealed, and the advantages of MPC over conventional grouting materials are clarified. The main conclusions are as follows:

(1) MPC exhibits excellent comprehensive performance as a sleeve grouting material, with superior fluidity, flexural strength of 54.0 MPa, compressive strength of 77.5 MPa, and tensile strength of 38.6 MPa, all of which meet the technical specifications for sleeve grouting materials. The connections satisfy Type I/II joint requirements in JGJ 107-2016, and the sleeve remains elastic throughout loading, providing sufficient safety margin. In terms of mechanical equivalence, the ultimate bond strength of MPC (18.73–35.00 MPa) is comparable to typical UHPC (16.62–35.47 MPa), with an ultimate bearing capacity difference of less than 5% and consistent failure mode evolution, confirming that MPC can fully utilize rebar strength and meet core mechanical requirements.

(2) MPC enables reliable load transfer and ideal failure mode control. Two failure modes are observed: rebar tensile fracture (8d anchorage length) and interface slip failure (shorter anchorage lengths). Increasing anchorage length and rebar strength grade significantly improves ultimate load and promotes the failure mode to transform into ideal rebar fracture. The ultimate load of the S400 series increases by 50.61% from 4d to 8d; at 6d anchorage length, the S600 series achieves a 44.85% higher load than the S400 series. Benefiting from high bonding efficiency, MPC achieves Type I/II performance at a 6d–8d anchorage length, supporting smaller sleeve size, lower material consumption, and more compact and efficient connection design.

(3) The bond strength of the MPC–rebar interface exhibits clear regularity. Average bond strength is negatively correlated with anchorage length and positively correlated with rebar strength grade. For HRB400E, bond strength decreases by 24.70% from 4d to 8d; increasing the rebar grade to HRB600E increases bond strength by 30.38–40.73% under the same anchorage length.

(4) Strain evolution and interface mechanisms are clarified. Before rebar yielding, sleeve and rebar strains increase linearly with load; sleeve strain rises from the grouting end to the middle. The circumferential strain at the sleeve end transfers from compression to tension at a specific load level, reflecting the constraint and stress transfer mechanism of the MPC-grouted sleeve.

(5) A bond strength calculation formula is fitted for slip failure, and the calculated values agree well with test results. Considering safety and construction tolerance, 8d is recommended as the critical anchorage length for HRB400E and HRB600E rebars in MPC-grouted sleeve connections. In terms of construction superiority, MPC features rapid curing (initial setting within 15–30 min) and excellent fluidity, which greatly shorten the construction period, improve grouting compactness and quality controllability, and enable efficient application in accelerated prefabricated construction.

(6) This study provides clear engineering implications for design and application. Using MPC grout and an anchorage length of 8d can realize ideal rebar tensile failure, meet the Type I/II joint requirements, fully utilize the strength of high-strength rebars, and ensure the safety, reliability and construction efficiency of prefabricated sleeve connections.

Several limitations of this study should be noted. The number of specimens is limited, with no repeated tests for each parameter group, which restricts the evaluation of data variability and may reduce the statistical universality of the conclusions. Nevertheless, the test results present clear and consistent regularity, and the key findings are reasonable and supported by the existing literature. Thus, the main conclusions on the mechanical performance and engineering potential of MPC grouted sleeve connections remain valid and reliable.

## Figures and Tables

**Table 1:** Mix ratio of MPC grouting material.

Material	Proportion of Ingredients/%		
	Polyol	Polyisocyanate	Cement
MPC	25	25	50

**Table 2:** Mechanical properties of MPC.

Numbers	MPC-T1	MPC-T2	MPC-T3	Mean	SD
Flexural strength (MPa)	51.1	56.8	54.2	54.0	2.3
Compressive strength (MPa)	75.3	78.9	78.3	77.5	1.6
Tensile strength (MPa)	36.3	39.4	40.1	38.6	1.7

**Table 3:** Specimen size.

Specimen	*d*/mm	*D_s_*/mm	*c*/mm	*D_m_*/mm	*L*/mm	*l_a_*
S400-MPC-4d	32	60	14	4	320	4*d*
S400-MPC-6d					450	6*d*
S400-MPC-8d					580	8*d*
S600-MPC-4d					320	4*d*
S600-MPC-5d					390	5*d*
S600-MPC-6d					450	6*d*
S600-MPC-8d					580	8*d*

Note: *d* and *l_a_* are the diameter and anchoring length of the rebar, respectively; *D_s_*, *D_m_* and *L* are the inner diameter, wall thickness and length of the sleeve, respectively; *c* is the thickness of the protective layer.

**Table 4:** Material properties of reinforcements.

Reinforcements	Diameter (mm)	*f_y_* (MPa)	*f_s_* (MPa)	Elongation After Breaking/%	Elastic Modulus (MPa)
HRB400E	32	464.2	608.0	18.5	2.06 × 10^5^
HRB600E	32	675.2	836.6	18.1	2.06 × 10^5^

**Table 5:** Test results and failure mode.

Specimen	*f_u_* (MPa)	*P_u_* (kN)	*f_u_* (*f_yk_*)	*f_u_* (*f_stk_*)	*τ_u_* (MPa)	*u*_0_ (mm)	Failure Mode
S400-MPC-4d	397.73	319.88	0.99	0.74	24.87	0.006	Sliding failure
S400-MPC-6d	518.48	416.98	1.29	0.96	21.61	0.001	Sliding failure
S400-MPC-8d	599.04	481.78	1.30	1.10	18.73	0.019	Tensile failure
S600-MPC-4d	560.10	450.21	0.93	0.77	35.00	0	Sliding failure
S600-MPC-5d	662.87	532.84	1.10	0.91	33.14	0.06	Sliding failure
S600-MPC-6d	751.04	603.72	1.25	1.04	31.29	0.09	Sliding failure
S600-MPC-8d	781.20	628.28	1.30	1.07	24.42	0.07	Tensile failure

Note: *f_u_* is the ultimate tensile strength; *P_u_* is the ultimate load; *f_yk_* and *f_s__tk_* are the standard yield and ultimate strength of steel bars, respectively; *τ_u_* is the average bond strength; *u*_0_ is the residual deformation.

**Table 6:** Performance comparison of MPC and UHPC grouts for grouted sleeve connections.

	Specimen	*f_y_*/MPa	*f_c_*/MPa	*d*/mm	*D_s_*/mm	*l_a_*	*P_u_*/kN	*τ_u_*/MPa	Failure Mode
Author	S400-MPC-4d	464.2	77.5	32	60	4d	319.88	24.87	Sliding
	S400-MPC-6d	464.2	77.5	32	60	6d	416.98	21.61	Sliding
	S400-MPC-8d	464.2	77.5	32	60	8d	481.78	18.73	Tensile
	S600-MPC-4d	675.2	77.5	32	60	4d	450.21	35.00	Sliding
	S600-MPC-5d	675.2	77.5	32	60	5d	532.84	33.14	Sliding
	S600-MPC-6d	675.2	77.5	32	60	6d	603.72	31.29	Sliding
	S600-MPC-8d	675.2	77.5	32	60	8d	628.28	24.42	Tensile
Fu [25]	S600-G1-4d	645.3	119.6	32	60	4d	449.11	34.92	Sliding
	S600-G1-5d	645.3	119.6	32	60	5d	523.54	32.56	Sliding
	S600-G1-6d	645.3	119.6	32	60	6d	529.74	27.46	Sliding
	S600-G1-8d	645.3	119.6	32	60	8d	674.14	26.21	Tensile
	S600-G2-4d	645.3	128.4	32	60	4d	456.22	35.47	Sliding
	S600-G2-5d	645.3	128.4	32	60	5d	533.93	33.21	Sliding
	S600-G2-6d	645.3	128.4	32	60	6d	603.57	31.29	Sliding
	S600-G2-8d	645.3	128.4	32	60	8d	674.75	26.23	Tensile
Zheng [26]	SM-SB-G1-D14	430	63	14	42	9.1d	94.1	16.80	Tensile
	SM-SB-G1-D14	430	63	14	42	9.1d	94.5	16.87	Tensile
	SM-SB-G1-D14	430	63	14	42	9.1d	93.1	16.62	Tensile
	SM-SB-G1-D16	440	63	16	42	8d	120.2	18.69	Tensile
	SM-SB-G1-D16	440	63	16	42	8d	121.9	18.96	Tensile
	SM-SB-G1-D16	440	63	16	42	8d	123.4	19.19	Tensile
	SM-SA-G1-D16	440	63	16	42	8d	124.5	19.36	Tensile
	SM-SA-G1-D16	440	63	16	42	8d	124.4	19.34	Tensile
	SM-SC-G2-D16	440	70.2	16	42	7d	119.4	21.22	Tensile
	SM-SC-G2-D16	440	70.2	16	42	7.1d	119.2	20.89	Tensile
	SM-SC-G2-D16	440	70.2	16	42	7.2d	121.1	20.92	Tensile
	SM-SD-G2-D22	452	70.2	22	50	7.2d	238.6	21.81	Tensile
	SM-SD-G2-D22	452	70.2	22	50	7.2d	248.3	22.69	Sliding
	SM-SD-G2-D22	452	70.2	22	50	7.2d	237.6	21.71	Tensile
	SM-SD-G2-D25	455	70.2	25	57	7.1d	299	21.46	Tensile
	SM-SD-G2-D25	455	70.2	25	57	6.9d	316	23.34	Sliding
	SM-SD-G2-D25	455	70.2	25	57	7d	300.8	21.90	Tensile
	SM-SD-G3-D25	455	75.6	25	57	7d	320.5	23.33	Sliding
	SM-SD-G3-D25	455	75.6	25	57	7.1d	300.1	21.54	Tensile

**Table 7:** The test values of the average bond strength compared to the calculated values.

Specimen	*f_c_* (MPa)	*τ_u_* (MPa)	*l_a_* (d)	*l_a,c_* (d)	*f*_y_ (MPa)	*τ_u,c_* (MPa)	*τ_u,c_*/*τ_u_*
S400-MPC-4d	77.5	24.87	4	4.5	464.2	25.99	1.05
S400-MPC-6d	77.5	21.61	6	5.9	464.2	19.69	0.91
S400-MPC-8d	77.5	18.73	8	7.2	464.2	16.16	0.86
S600-MPC-4d	77.5	35.00	4	4.4	675.2	38.24	1.09
S600-MPC-5d	77.5	33.14	5	5.1	675.2	32.82	0.99
S600-MPC-6d	77.5	31.29	6	5.8	675.2	28.96	0.93
S600-MPC-8d	77.5	24.42	8	7.1	675.2	23.78	0.97

## Data Availability

The original contributions presented in this study are included in the article. Further inquiries can be directed to the corresponding author.

## References

[1] Xu Z., Zhang Z., Xu T. (2021). State of the Art on Prefabricated Concrete Bridge Structures in 2020. J. Civ. Environ. Eng..

[2] Moosavi M., Jafari A., Khosravi A. (2005). Bond of Cement Grouted Reinforcing Bars under Constant Radial Pressure. Cem. Concr. Compos..

[3] Xiao S., Fomin N.I., Liu C., Yang H. (2025). Systematic Review of High-Performance Grouting Materials for Prefabricated Grouted Sleeve Connections in Building Structures. Results Eng..

[4] Ling J.H., Rahman A.B.A., Ibrahim I.S. (2014). Feasibility Study of Grouted Splice Connector under Tensile Load. Constr. Build. Mater..

[5] Li P., Huang M., Quan X., Kuang Y., Chen F., Liu B., Li H. (2025). Experimental and Numerical Investigation into the Tensile Performance of a Novel Self-Adaptive Grouting Evolved Sleeve. Structures.

[6] Yang Z., Xu W., Wang J., Qian Z., Pan K., Zhou D., Chen Y., Huang Y., Zheng Y. (2025). Seismic Performance of Precast Roof and Sidewall Joints for Underpass with UHPC-Alveolus-Grouting Sleeve Composite Connection. Structures.

[7] Gao X., Li Z. (2019). Study on Embedded Length of High-Strength Reinforcement in Splice Sleeve. J. Huazhong Univ. Sci. Technol..

[8] Liu L., Xiao J., Ding T., Zhang K. (2021). Test and Simulation on Bond Behavior between Sleeve Grout and High Strength Steel Rebar. J. Tongji Univ..

[9] Wang Z., Zhu J., Wang J., Zhao G., Sun S., Zhang J. (2021). Experimental Study on a Novel UHPC Grout-Filled Pipe Sleeve with Mechanical Interlocking for Large-Diameter Deformed Bars. Eng. Struct..

[10] Seo S.-Y., Nam B.-R., Kim S.-K. (2016). Tensile Strength of the Grout-Filled Head-Splice-Sleeve. Constr. Build. Mater..

[11] Einea A., Yamane T., Tadros M.K. (1995). Grout-Filled Pipe Splices for Precast Concrete Construction. PCI J..

[12] Zhao G., Liu L., Wang M., Zhao Q., Yang Z., Song J., Cheng B. (2025). Effects of Grouting Defects on the Mechanical Performance of Half-Grouted Connections at Low and Normal Temperatures. Mater. Struct..

[13] Wu X., Feng L., Wang T. (2013). Experimental Research on Effects of Grout Age and Types of Steel Bars on Mechanical Behavior of Grout Sleeve Splicing for Reinforcing Bars. Build. Struct..

[14] Yu Q., Zhang Y., Gong X., Bai S., Fan B. (2017). Experimental Study of Grouted Sleeve Lapping Connector under Tensile Load. J. Croat. Assoc. Civ. Eng..

[15] Yu Q., Sun J., Yuan W. (2018). Experimental Study on Bonding Properties between Ribbed Steel Bars and Grouting Material Constrained by Type II APC Sleeve. J. Harbin Inst. Technol..

[16] Cao X., Peng J.C., Jin L.Z. (2014). Experimental research on mechanical performance of prestressed RPC beam. J. Wuhan Univ. Technol..

[17] Long Q., Ding M., Huang Z., Ke W., Hu Z. (2025). Research Status and Prospects of Grouted Sleeve Connections in Prefabricated Structures. Buildings.

[18] Liu L., Ouyang X., Wang X., Zhang Q. (2025). Multi-Factors Effects on Developments of Bond Behaviors between the High-Strength Grout and Steel Rebar and Its Optimization. Structures.

[19] Zou B., Yin J., Cao C., Long X. (2025). Mechanical Performance Analysis of Rubber Elastic Polymer-Polyurethane Reinforced Cement-Based Composite Grouting Materials. J. Polym. Mater..

[20] (2001). Test Specification for Polymer Modified Cement Mortar.

[21] (2019). Test Methods of Physical and Mechanical Properties of Concrete.

[22] (2019). Cementitious Grout for Sleeve of Rebar Splicing.

[23] (2015). Technical Specification for Application of Reinforcement Sleeve grouting Connection.

[24] (2016). Technical Specification for Reinforcement Mechanical Connection.

[25] Fu T., Ren X., Li Y., Wang K., Zhu Z. (2022). Experimental Study on the Mechanical Behavior of HTRB600E Steel Sleeve Grouting Based on UHPC. Case Stud. Constr. Mater..

[26] Zheng Y.F. (2015). Research on Rebar Splicing System by GDPS Grout-Filled Coupling Sleeve. Ph.D. Thesis.

